# Supplementary material for: Development and Validation of Benzophenone Derivatives in Packaged Cereal-Based Foods by Solid–Liquid Extraction and Ultrahigh-Performance Liquid Chromatography–Tandem Mass Spectrometry
Source: Foods. 2022 May 7;11(9):1362. doi: 10.3390/foods11091362 (PMC9103691; doi:10.3390/foods11091362)
Supplement: Supplementary file 1 [file foods-11-01362-s001.zip › foods-1688077-supplementary.pdf]

Supplementary materials

Development and validation of benzophenone derivatives in packaged cereal-based foods by solid-liquid extraction and ultrahigh-performance liquid chromatography–tandem mass spectrometry

Yu-Fang Huang [1,2,3,*], Jun-Jie Huang [1], Xuan-Rui Liu [1]

[1] Department of Safety, Health and Environmental Engineering, National United University, Miaoli 36063, Taiwan; k10220202@gmail.com (J.-J.H.); aass1aass35@gmail.com (X.-R.L.)
[2] Center for Chemical Hazards and Environmental Health Risk Research, National United University, Miaoli 36063, Taiwan
[3] Institute of Food Safety and Health Risk Assessment, National Yang Ming Chiao Tung University, Taipei 11221, Taiwan

[*]Correspondent: Dr. Yu-Fang Huang

Department of Safety, Health and Environmental Engineering, National United University,

Miaoli, Taiwan

Tel: 886-37-382272; Fax: 886-37-382281

Email: yfh@nuu.edu.tw

Total number of figures:2

(A)

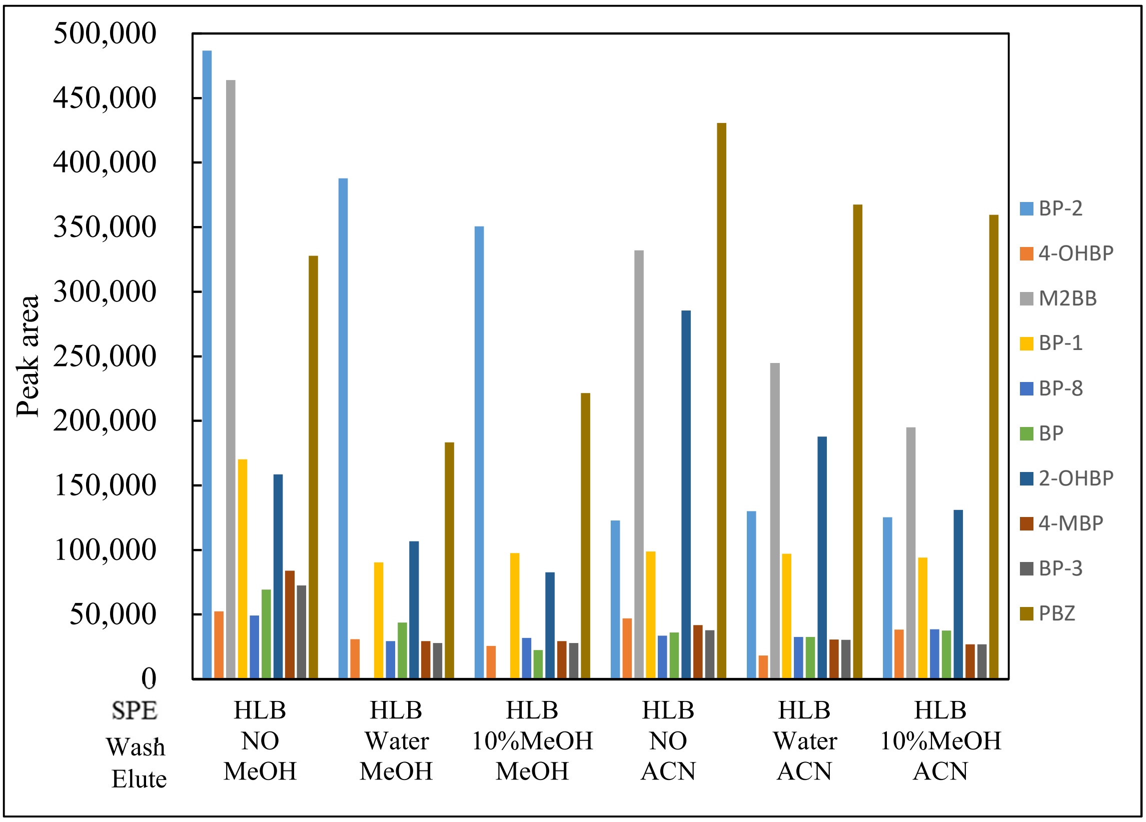

(B)

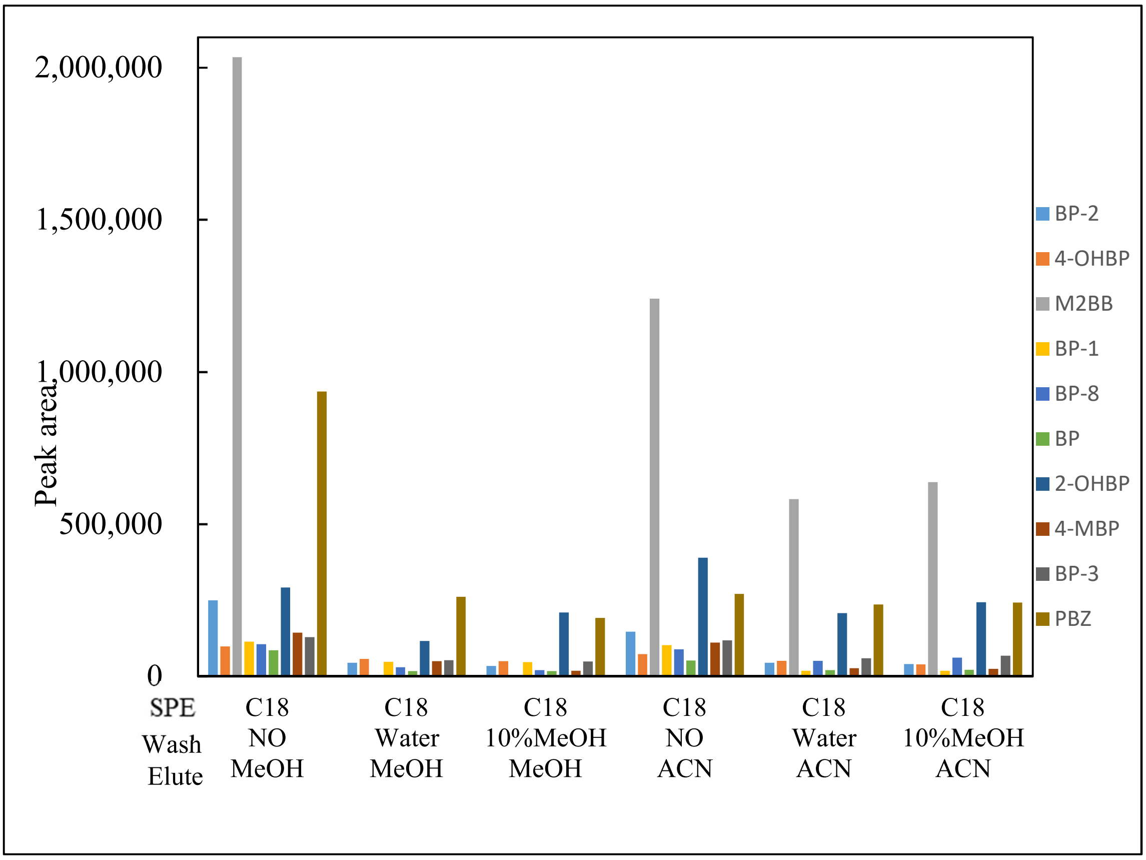

Figure S1 Comparison of the peak areas for BPs standards and IS (20 and 8 ng/g) across the cartridges with (A) HLB-SPE and (B) C18-SPE in three wash conditions (no wash, water, 10% MeOH) and elution solvents in MeOH and ACN (n=5)

(A)

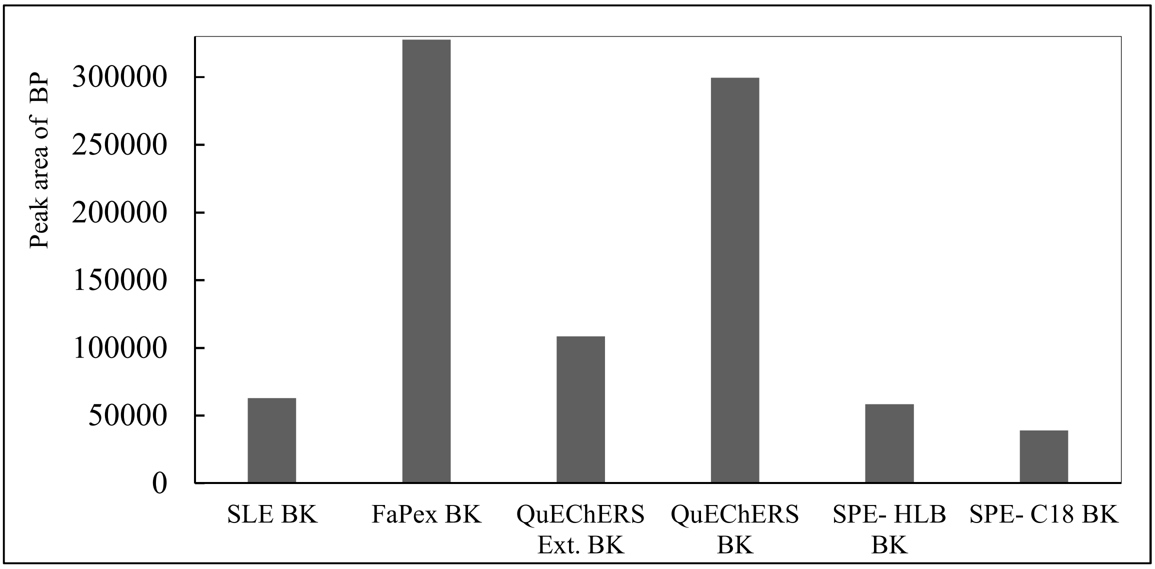

(B)

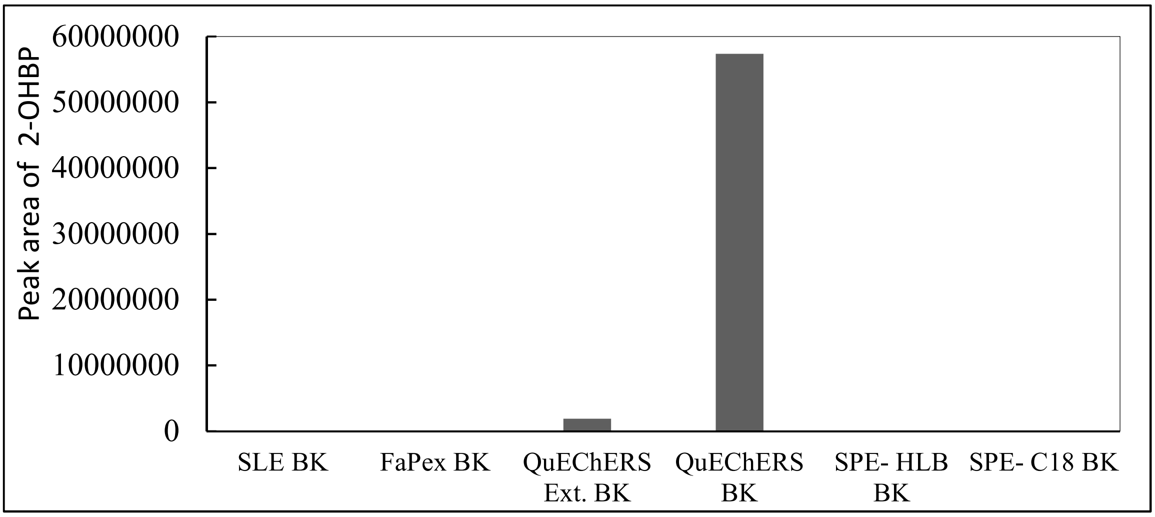

Figure S2 Comparison of the peak areas for (A) BP and (B) 2-OHBP across six pretreatment methods in blank pastry sample.
